# Intracranial Empyema in Children: A Single-center Retrospective Case Series

**DOI:** 10.1097/INF.0000000000004064

**Published:** 2023-10-11

**Authors:** James J. Gilchrist, Tom Hoy, Else M. Bijker, Emily A. Lees, Laura Wilkins, Madeleine Oliver, Dominic F. Kelly, Stéphane C. Paulus, Amedeo Calisto

**Affiliations:** From the * Department of Paediatrics, University of Oxford, Oxford, United Kingdom; † Department of Paediatric Neurosurgery, Oxford University Hospitals NHS Foundation Trust, Oxford, United Kingdom; ‡ Department of Paediatrics, Maastricht University Medical Center, Maastricht, The Netherlands; § Fitzwilliam College, Cambridge, United Kingdom; ¶ Oxford Foundation School, Health Education England – Thames Valley, Oxford, United Kingdom; ‖ South Thames Foundation School, Health Education England, London, United Kingdom; ** NIHR Oxford Biomedical Research Centre, John Radcliffe Hospital, Oxford, United Kingdom.

**Keywords:** empyema, subdural, extradural, infection, *anginosus*

## Abstract

We conducted a retrospective, observational study of 42 children with intracranial empyema admitted to a pediatric neurosurgical center over a 9-year period. Intracranial empyema is rare, but causes significant morbidity and mortality. Twenty-eight cases had neurosurgical source control, more commonly for subdural collections. *Streptococcus anginosus* group bacteria are important pathogens in subdural empyema, whose isolation predicts more complicated postoperative courses.

Intracranial empyema, the accumulation of pus in the extradural or subdural space, results from direct extension of local infection, most commonly sinusitis or otitis media/mastoiditis or hematogenous spread. Both extradural empyema (EDE) and subdural empyema (SDE) are rare, with a combined incidence of 2.8–5.7 per million children per year.^1,2^ SDE in infants typically follows bacterial meningitis, whereas empyema in older children more commonly complicates ear or sinus infections. SDE can result in catastrophic neurological insult, with long-term neuro-disability reported in 15%–35% and mortality in 4%.^3^

SDE-associated mortality in the preantibiotic era approached 100%,^4^ and remains over 10% in resource-poor settings.^5^ The drivers of improved outcomes for intracranial empyema are likely to be multifactorial. A clear understanding of current clinical practice, as well as better-defining children who have poor outcomes, will inform clinical practice guidelines. Here we sought to characterize the clinical and microbiologic features of these infections by reviewing our experience with intracranial empyema in children over a 9-year period.

## METHODS

We reviewed the clinical notes of children (<18 years) with intracranial empyema admitted to Oxford University Hospitals, Oxford, United Kingdom between January 1st, 2013 and January 31st, 2022. Methods are described in full online (see Methods, Supplemental Digital Content 1, http://links.lww.com/INF/F200).

## RESULTS

An extended description of the demographics, laboratory investigations, underlying comorbidities and management is provided online (see Results and Discussion, Supplemental Digital Content 2, http://links.lww.com/INF/F201).

### Demographics and Etiology

**FIGURE 1.**
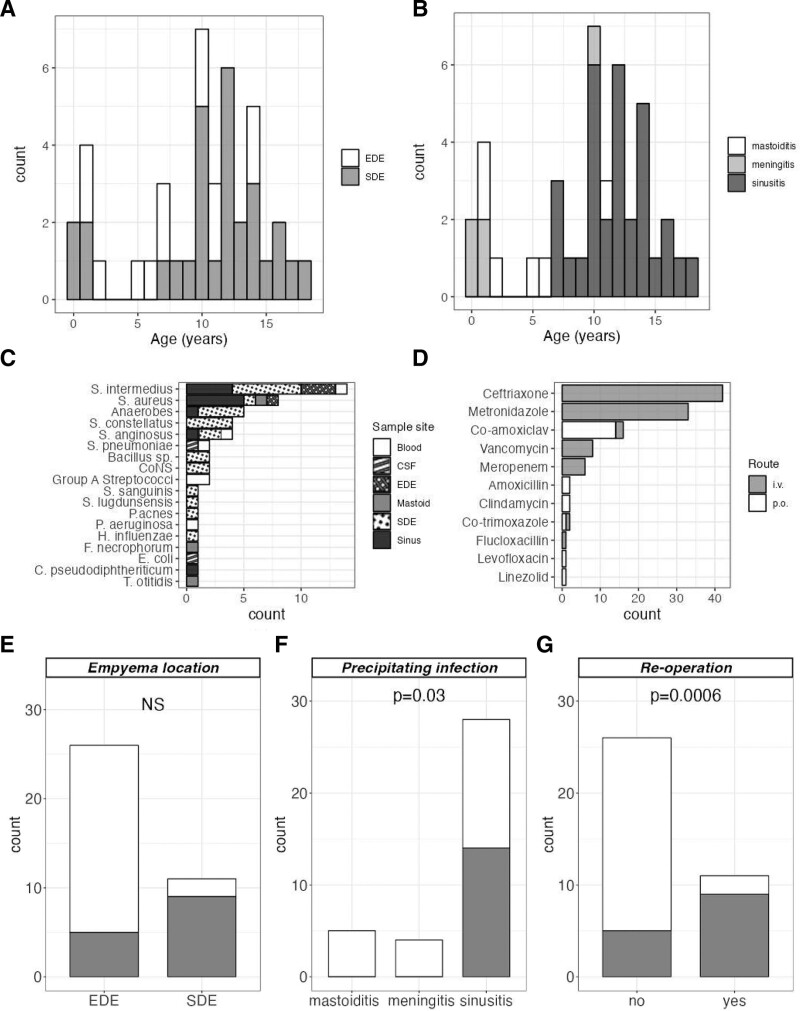
Demographics and microbiology of intracranial empyema in children. Age distribution of children (n = 42) with intracranial empyema, colored according to the site of empyema (A) and precipitating infection (B). C: Microbiology of empyema in children: isolates are colored/patterned accorded to sample site. D: Antibiotic use among children with empyema; intravenous (i.v.), oral (p.o.). E: Comparison of the proportion of children with *Streptococcus anginosus* group (SAG) *Streptococci* isolated at operation by empyema location. F: Comparison of the proportion of children with SAG *Streptococci* isolated at operation by precipitating infection. G: Comparison of the proportion of children with SAG *Streptococci* isolated at operation by subsequent need for reoperation. Cases in which SAG *Streptococci* were isolated are highlighted (gray). The association between SAG *Streptococci* and subsequent reoperation is independent of the site of empyema (Mantel–Haenszel OR = 8.2, *P* = 0.020), and precipitating infection (Mantel–Haenszel OR = 9.8, *P* = 0.018). EDE indicates extradural empyema; OR, odds ratio; SDE, subdural empyema.

During the study period, 42 children were admitted with a diagnosis of community-acquired intracranial empyema; 13 EDE, 29 SDE. The estimated risk of intracranial empyema among children during the study period was of 4.9 per 1,000,000 per year. The median age was 10.5 years (range 3 months–17.8 years), showing a bimodal distribution, with peaks at 10 months and 11 years (Fig. 1A). All intracranial empyema were complications of identifiable primary infection (Fig. 1B); sinusitis (n = 31, 74%), mastoiditis (n = 6) and meningitis (n = 5).

### Clinical Presentation

Children commonly presented with fever (n = 36, 88%) and headache (n = 31, 79%). Seizures, focal neurological signs, reduced Glasgow Coma Scale and vomiting were also common (see Table, Supplemental Digital Content 3, http://links.lww.com/INF/F202), but each were present in less than half of children with empyema. Vomiting, reduced Glasgow Coma Scale and focal neurology were all more common in children with SDE than with EDE. Children with empyema had a median interval from the onset of symptoms to their first hospital admission of 7 days (range 0–36 days).

### Surgical Management

Twenty-eight children underwent surgical source control, more commonly among children with SDE (odds ratio = 10.4, *P* = 0.001). Twenty-six of 28 children underwent their operation on the date of admission/abnormal imaging or the following day. Of the 14 children not undergoing neurosurgical intervention, 9 (3 SDE and 6 EDE) had sinus washout or mastoidectomy alone and 5 children (2 SDE, 3 EDE) had no intracranial or extracranial source control. Children selected for conservative management had no neurological deficit and had small, shallow intracranial collections (median depth 7 mm).

### Microbiology and Antibiotic Management

Bacteria were isolated from blood (n = 6), cerebrospinal fluid (n = 2), pus isolated at sinus/mastoid surgery (n = 15) and subdural (n = 25) and extradural (n = 4) pus during empyema evacuation. A total of 52 bacteria were isolated from 24 children, including 18 distinct organisms (Fig. 1C; see Table, Supplemental Digital Content 4, http://links.lww.com/INF/F203). At least 6 of these bacteria, isolated from empyema, sinus or mastoid sampling, are skin and upper respiratory tract commensals (coagulase-negative *Staphylococci*, *Corynebacterium pseudodiphtheriticum*, *C. acnes*, *S. sanguinis* and *Turicella* sp.) or common environmental contaminants (*Bacillus* sp.), which may represent contamination. Specimens from 18 children were sterile (3 managed conservatively). Molecular methods were not routinely used to improve diagnostic yield in culture-negative samples during the study period. Samples from blood and cerebrospinal fluid yielded a single pathogen, whereas pus from sinus surgery or empyema commonly yielded more than 1 pathogen: 7 polymicrobial empyema samples and 2 polymicrobial sinus samples.

We used combination therapy ceftriaxone and metronidazole as empiric cover for suppurative intracranial infection. Initial therapy was intravenous, with the early transition to oral metronidazole as soon as the enteral route was appropriate. In practice, 11 antimicrobials were used to treat children in this study (Fig. 1D). Ceftriaxone and metronidazole in combination was the most common empiric antibiotic choice at admission to hospital (n = 21).

The median total antibiotic duration was 47 days (range 28–130). In 16 cases, after a course of intravenous therapy, antibiotics were switched to an oral agent for the remainder of the treatment course. The oral agent was most commonly coamoxiclav (n = 11), with conversion at a median of 33 days (range 10–53). We selected children for oral switch where there was adequate source control, down-trending or normalized inflammatory markers, and no clinical features suggestive of disease recurrence. This decision was supported by imaging in 9 children. Conversion to oral antibiotics shortened the duration of intravenous antibiotics by a median of 13 days (*P* = 0.002) and had no effect on overall antibiotic duration.

### Complications and Outcome

Intracranial empyema was complicated by venous sinus thrombosis in 9 children (21%), cerebral infarct in 5 (12%), intracranial abscess in 5 (12%) and osteomyelitis in 12 (29%; see Table, Supplemental Digital Content 3, http://links.lww.com/INF/F202). One child with SDE secondary to sinusitis died. At discharge, 9 children (23%, all with SDE) had weakness, with weakness persisting to the most recent follow-up in 4 (10%) children. More subtle neurocognitive changes, for example, behavior change, sleep difficulties and difficulties with word-finding, memory and concentration, were documented in a further 8 (21%) children at the last follow-up. Outcomes in all conservatively managed cases were favorable.

### *Streptococcus anginosus* Group Bacteria

Of 37 children with intracranial empyema in whom microbiological sampling was performed, 14 children (38%) had *Streptococcus anginosus* group (SAG; *S. anginosus*, *S. intermedius* or *S. constellatus*) bacteria isolated. The majority of these (n = 13) were isolated from children with SDE (Fig. 1E). All children with SAG *Streptococci* had empyema secondary to sinusitis (Fig. 1F). Isolation of SAG *Streptococci* predicted the need for reoperation in children with empyema (Fig. 1G). Among children with SAG *Streptococci*, 9 of 14 (64%) required reoperation, compared with 3 of 23 (13%) among children in whom SAG *Streptococci* was not isolated (odds ratio = 11.5, *P* = 0.003). Many children undergoing reoperation in the context of SAG *Streptococci* did so because of reaccumulation of empyema/inadequate source control (n = 7), with 1 child needing intervention for raised intracranial pressure and 1 child experiencing late bone flap osteomyelitis.

## DISCUSSION

Here we describe 42 children with intracranial empyema presenting to a single United Kingdom center over a 9-year period. Intracranial empyema are rare but important complications of common childhood infections. They affect young infants and older children, have nonspecific clinical presentations, and frequently have poor outcomes. We identify SAG *Streptococci* as predictors of complicated postoperative courses in these children.

The bimodal age distribution of disease that we observe has been consistently reported for both children’s brain abscess^1,2^ and intracranial empyema.^6^ This reflects the age distribution of precipitating infections; meningitis and mastoiditis causing disease in younger children, and sinusitis doing so in older children. In keeping with previous studies,^1,2,6^ children presented commonly with fever and headache, but had the clinical triad of fever, headache and focal neurologic signs in only a quarter of cases.

In our study, 1 child with sinogenic SDE died. This is similar to the reported mortality of children with brain abscess in the United Kingdom (5.9% and 3.2%)^1,2^ and mortality reported in children with intracranial empyema (4%).^3^ While mortality was uncommon, 4 children (10%) had focal neurological deficits persistent to the most recent clinic follow-up. There is considerable heterogeneity in reported long-term morbidity following brain abscess and empyema in children, with estimates in the published literature ranging between 12% and 35%.^1,7^ This heterogeneity is very likely to be secondary to small sample sizes and inconsistent definitions of what constitutes long-term morbidity in these children. Indeed, in our own data, while only 4 children had persistent focal neurologic deficits, 8 (21%) had more subtle, long-term, neurocognitive difficulties and it seems likely that the published data represents an under-estimate of the true long-term morbidity associated with this condition. A further limitation of the published literature and our data is that they reflect practice and outcomes in large, highly-specialized pediatric neurosurgical centers, and these outcomes are unlikely to be representative of those in resource-limited settings.^5^

Finally, isolation of SAG *Streptococci* was significantly associated with increased risk of reoperation. This expands on the previously reported observation that SAG *Streptococci* are associated with the development of intracranial complications, including intracranial empyema, in children with sinusitis,^8^ and complicated postoperative courses requiring reoperation in patients with appendicitis.^9,10^ Moreover, in a recent case series of SDE, SAG *Streptococci* were isolated in the majority (6 of 7, 86%) of children requiring reoperation, compared with 20 of 29 (69%) not requiring repeat surgery, however, this difference was not statistically significant.^6^

Despite considerable improvements in outcome following intracranial empyema in children over the last 50 years, our study highlights the ongoing burden of morbidity and mortality associated with this condition. We identify that children with SAG *Streptococci* infections are more likely to require repeat operative intervention. Future studies should leverage these data to prospectively evaluate modifications to the management of children with intracranial empyema secondary to SAG *Streptococci*.

## Supplementary Material


