# Supplementary material for: Intracranial Empyema in Children: A Single-center Retrospective Case Series
Source: Pediatr Infect Dis J. 2023 Oct 11;42(11):e417–20. doi: 10.1097/INF.0000000000004064 (PMC10569674; doi:10.1097/INF.0000000000004064)
Supplement: Supplementary file 1 [file inf-42-e417-s001.pdf]

SDC 1. Methods

We identified children presenting over a 9-year period, between 1st January 2013 and 31st January 2022, with a hospital discharge ICD-10 code G062 (Extradural and subdural abscess, unspecified). The pediatric neurosurgical service at OUH provided care to a population of 942,879 children <18 years at the study mid-point (https://www.ons.gov.uk), which we used to calculate the estimated annual risk of intracranial empyema in children over the study period in this setting.

We used t-tests and ANOVA to compare continuous data between groups. Following a significant ANOVA we used Tukey post-hoc tests to make between groups comparisons. We assessed normality of continuous data using Shapiro-Wilk's tests, transforming data not normally distributed. Where transformation failed to normalise data we used Mann-Witney U tests and Kruskall-Wallis tests to compare between groups. We compared proportions between groups using Fisher exact tests, and used Mantel-Haenszel tests to compare proportions stratified by confounding covariates. Statistical analysis was performed in R.
