# Supplementary material for: Intracranial Empyema in Children: A Single-center Retrospective Case Series
Source: Pediatr Infect Dis J. 2023 Oct 11;42(11):e417–20. doi: 10.1097/INF.0000000000004064 (PMC10569674; doi:10.1097/INF.0000000000004064)
Supplement: Supplementary file 2 [file inf-42-e417-s002.pdf]

SDC2. Extended Results & Discussion

Demographics

Twenty-seven of 42 (64%) children with empyema were male. Children with extradural empyema were younger (median 7.2 years) than children presenting with subdural collections (median 11.7 years, p=0.019, SDC2 Figure 1A). There was some evidence for seasonality in the risk of empyema, with disease being more common between October and March than in the summer months (incidence rate ratio=1.83, p=0.05, SDC2 Figure 1B). The bimodal age distribution of empyema in children appears to be secondary to the age distribution of these index infections: children with empyema secondary to sinusitis were significantly older (SDC2 Figure 1C) than children with mastoiditis (p=0.0004) or meningitis (p=0.0002).

Timing of admission

Children with empyema had a median interval from onset of symptoms to their first hospital admission of 7 days (range 0 to 36 days). Time to admission did not differ between children with EDE and SDE (SDC2 Figure 1D). Children presenting with empyema secondary to meningitis had significantly shorter times to presentation (median 0.5 days, range 0 to 3 days) as compared to children with mastoiditis (p=0.02) or sinusitis (p=0.0002). This may simply reflect that parents and primary care physicians are more likely to seek hospital care for undifferentiated fever in young infants. Importantly however, time to admission was not associated with increased complications or poor outcome.

Inflammatory markers

At admission, plasma CRP levels were raised (>5mg/L) in 39 of 41 children, and white cell counts were raised ($>11.0x10^9$/L) in 36 of 40 children. The median admission CRP was 155mg/L (range 2.2-318mg/L), and the median WCC was $16.3x10^9$/L (range $3.8-43.0x10^9$/L). Both CRP ($p=8.81x10^{-5}$, SDC2 Figure 2A) and white cell count (p=0.012, SDC2 2B) were higher at admission in children with SDE. Inflammatory markers at admission did not differ with precipitating infection.

Comorbidity

The majority of children with intracranial empyema had no underlying medical conditions. Among children with empyema secondary to sinusitis, one child had a history of chronic rhinosinusitis and another had had a previous episode of pelvic osteomyelitis. Two infants (aged 3 and 6 months) with SDE secondary to meningitis were ex-preterm: born at 28 and 30 weeks. There was a history of minor head trauma preceding presentation in 3 children.

Bony anatomical defects were uncommon in this cohort, with two children having defects identified; one tegmen defect resulting in extradural empyema secondary to mastoiditis, and one frontal bone defect resulting in extradural empyema secondary to sinusitis. It has not been our practice to systematically investigate children with intracranial empyema for underlying immunodeficiency. In this cohort, immunological evaluation was undertaken in 11 children. In all cases, that evaluation consisted of assessment of antibody immunity (immunoglobulin levels and vaccine responses), with additional testing for complement function (n=6), lymphocyte composition (n=10) and function (n=2), CD62L shedding (n=3) and neutrophil function (n=3) in a subset.

One child with SDE secondary to pneumococcal meningitis had an undefined antibody immunity defect, with persistently poor responses to pneumococcal immunisation with normal total immunoglobulin levels. This is consistent with data that children with invasive pneumococcal disease in the post-vaccine era are enriched for primary immunodeficiencies[1], and supports the targeted assessment of immune function in children whose presentation is otherwise suggestive of immunodeficiency, rather than the systemic evaluation of all children with intracranial empyema.

Surgical management

In children undergoing evacuation of their empyema, craniotomy was the most common surgical approach (SDC2 Figure 3A). Frontal sinus cranialisation was performed in 9 children, and additional sinus/mastoid surgery was performed alongside neurosurgical intervention in 14 cases. There were 11 cases requiring re-operation following initial source control. This was most frequently to achieve improved source control of intracranial pus (n=8), but also for external ventricular drain insertion (n=1) and bone flap osteomyelitis (n=2). The interval to re-operation for bone flap osteomyelitis was significantly longer than for re-accumulation of pus (p=0.008) or raised ICP (p=0.03, SDC2 Figure 3B).

SDC2. Figure 1

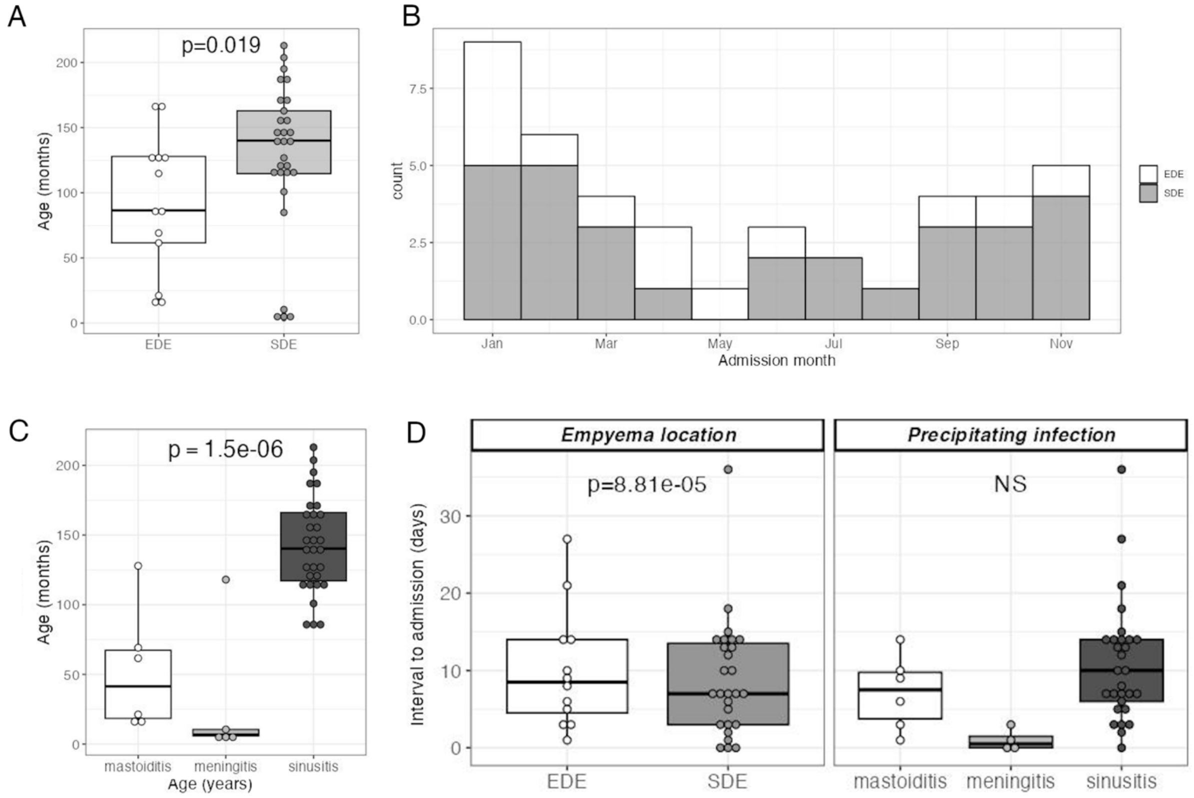

Demographics and time to admission of intracranial empyema in children. (A) Ages of children (n=42) with extradural and subdural empyema. (B) Seasonality of intracranial empyema in children. (C) Ages of children with empyema secondary to mastoiditis, meningitis and sinusitis. Children with mastoiditis (p=0.0004) and meningitis (p=0.0002) are significantly younger than are children with sinusitis. There is no significant difference in the ages of children with empyema secondary to mastoiditis and meningitis. (D) Comparison of interval (days) between first symptoms of disease and hospital admission by empyema location (left) and precipitating infection (right). Children with empyema secondary to meningitis have shorter interval to admission that do children with empyema secondary to mastoiditis (p=0.02) or sinusitis (p=0.0002). NS, not significant.

SDC2. Figure 2

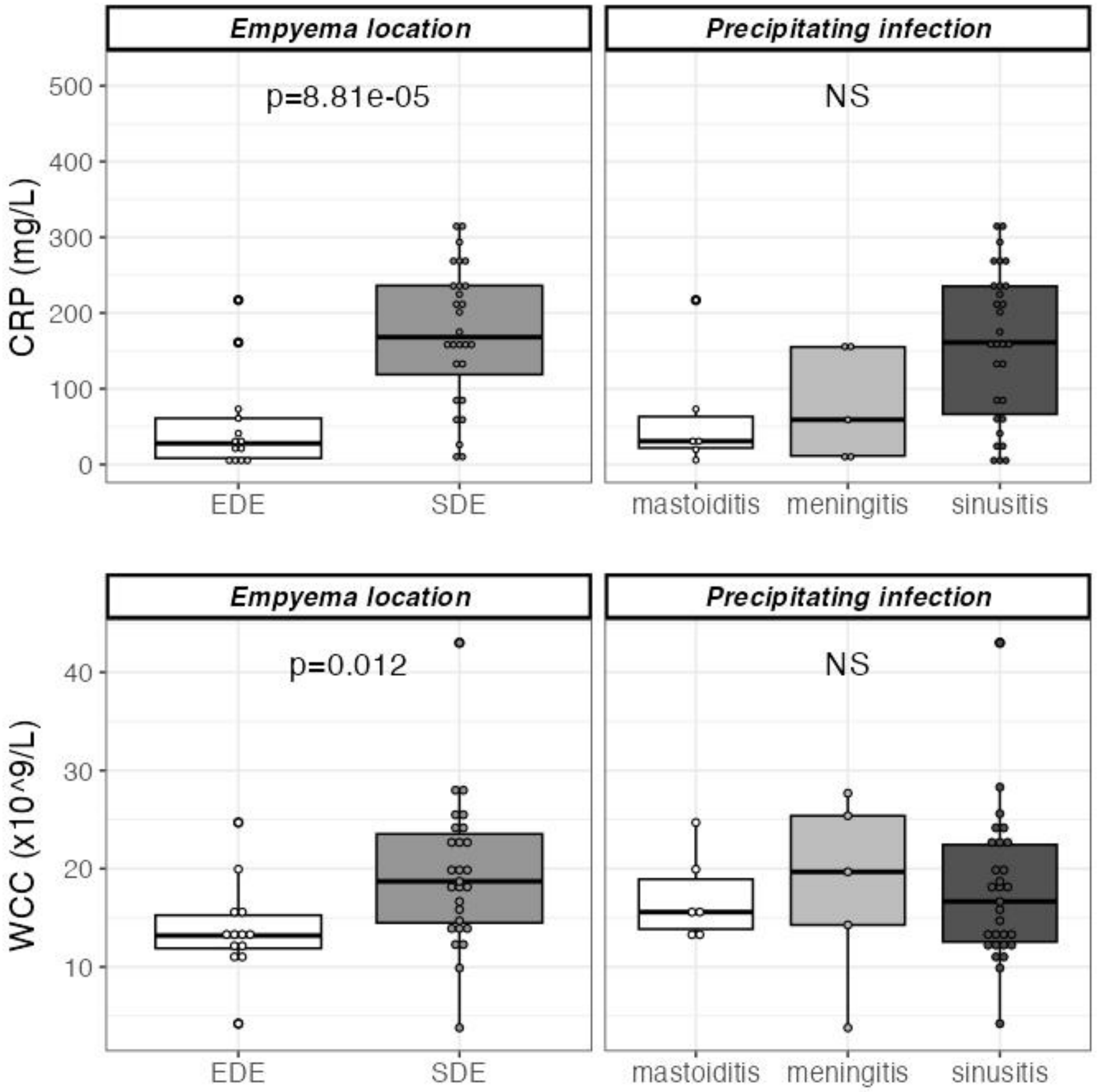

Inflammatory response of children with intracranial empyema. (A) Comparison of plasma CRP at admission between first symptoms of disease and hospital admission by empyema location (left) and precipitating infection (right). (B) Comparison of plasma CRP at admission between first symptoms of disease and hospital admission by empyema location (left) and precipitating infection (right). NS, not significant.

SDC2. Figure 3

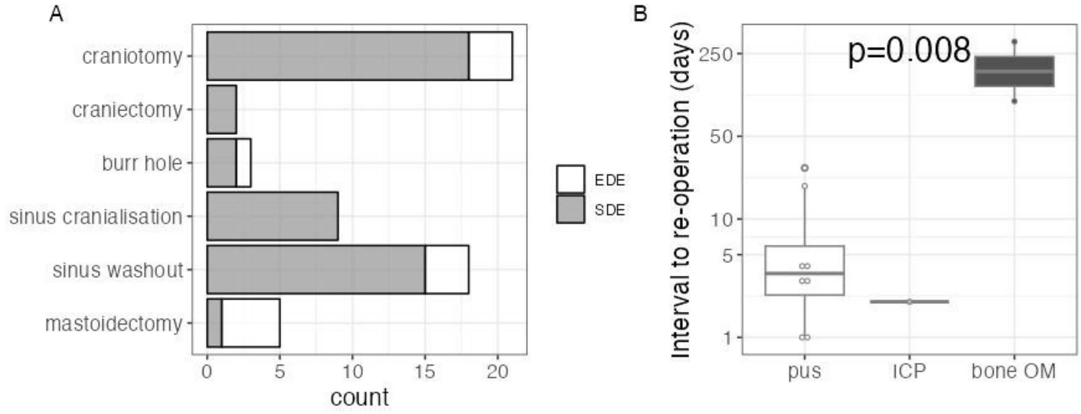

Surgical management of children with intracranial empyema. (A) Surgical procedures performed on children with intracranial empyema, coloured according to site of empyema: extradural (EDE, white), subdural (SDE, grey). (B) Interval (days) between first neurosurgical procedures and re-operation. Children with bone flap osteomyelitis have longer intervals to re-operation that do children re-operated for improved source control (pus, p=0.008) or raised intracranial pressure (ICP, p=0.03).

SDC2. References

1. Gaschignard J, Levy C, Chrabieh M, et al. Invasive pneumococcal disease in children can reveal a primary immunodeficiency. *Clin Infect Dis*. 2014;59(2):244–251.
