# Supplementary material for: Intracranial Empyema in Children: A Single-center Retrospective Case Series
Source: Pediatr Infect Dis J. 2023 Oct 11;42(11):e417–20. doi: 10.1097/INF.0000000000004064 (PMC10569674; doi:10.1097/INF.0000000000004064)
Supplement: Supplementary file 3 [file inf-42-e417-s003.pdf]

SDC3. Table

	Total (n=42)	SDE (n=29)	EDE (n=13)	p-value
Clinical Presentation				
Fever	36 (88%)	25 (86%)	11 (92%)	NS
Seizures	13 (32%)	10 (34%)	3 (25%)	NS
Headache	31 (79%)	24 (89%)	7 (58%)	NS
Vomiting	15 (37%)	14 (48%)	1 (8%)	0.01
GCS<15	16 (39%)	15 (52%)	1 (8%)	0.03
Focal neurology	13 (32%)	13 (45%)	0	0.007
Meningism	8 (20%)	8 (28%)	0	NS
Nasal discharge	15 (37%)	13 (45%)	2 (17%)	NS
Frontal swelling	10 (24%)	6 (21%)	4 (31%)	NS
Mastoid swelling	4 (10%)	0	4 (31%)	0.006
Complications				
Venous sinus thrombus	9 (21%)	5 (17%)	4 (31%)	NS
Infarct	5 (12%)	5 (17%)	0	NS
Intracerebral abscess	5 (12%)	4 (14%)	1 (8%)	NS
Osteomyelitis	12 (29%)	4 (14%)	8 (62%)	0.003
Outcome				
Mortality	1 (2%)	1 (3%)	0	NS
Weakness*	9 (23%)	9 (33%)	0	0.019
Weakness†	4 (10%)	4 (15%)	0	NS
Neurocognitive deficit†	8 (21%)	7 (27%)	1 (8%)	NS
SNHL†	2 (5%)	2 (8%)	0	NS
Epilepsy†	2 (5%)	1 (4%)	1 (8%)	NS

*At discharge.

†At most recent follow-up (10 months, range 1 to 74 months).

P-values compare proportions in subdural and extradural empyema cases. P-values are calculated with Fisher exact tests.

SDE, subdural empyema; EDE, extradural empyema; SNHL, sensorineural hearing loss.
