# Supplementary material for: Intracranial Empyema in Children: A Single-center Retrospective Case Series
Source: Pediatr Infect Dis J. 2023 Oct 11;42(11):e417–20. doi: 10.1097/INF.0000000000004064 (PMC10569674; doi:10.1097/INF.0000000000004064)
Supplement: Supplementary file 4 [file inf-42-e417-s004.pdf]

SDC4. Table

		Site		Precipitating infection			Source of sample				
	Total (n=42)	SDE (n=29)	EDE (n=13)	Mastoiditis	Meningitis	Sinusitis	Intracranial pus	Sinus	Mastoid	Blood	CSF
Microbiology											
Sterile	18	10	8	3	2	13					
Polymicrobial	9	9	0	0	0	9	7	2	0	0	0
Organisms											
S. intermedius	10	9	1	0	0	10	9	4	0	1	0
S. aureus	7	5	2	1	0	6	2	5	1	0	0
Anareobes	5	4	1	0	0	5	4	1	0	0	0
S. constellatus	4	4	0	0	0	4	4	0	0	0	0
S. anginosus	2	2	0	0	0	2	2	1	0	1	0
S. pneumoniae	1	1	0	0	1	0	0	0	0	1	1
Bacillus sp.	2	2	0	0	0	2	2	0	0	0	0
CoNS	2	2	0	0	0	2	2	0	0	0	0
Group A Streptococcus	2	2	0	0	0	2	0	0	0	2	0
C. pseudodiphtheriticum	1	1	0	0	0	1	0	1	0	0	0
E. coli	1	1	0	0	1	0	0	0	0	0	1
F. necrophorum	1	0	1	1	0	0	0	0	1	0	0
H. influenzae	1	1	0	0	0	1	1	0	0	0	0
P. aeruginosa	1	1	0	0	1	0	0	0	0	1	0
C. acnes	1	1	0	0	1	0	1	0	0	0	0
S. lugdunsensis	1	1	0	0	0	1	1	0	0	0	0
S. sanguinis	1	1	0	0	0	1	1	0	0	0	0
T. otitidis	1	0	1	1	0	0	0	0	1	0	0

Sampling at sinus or mastoid surgery yielded the sole positive microbiological isolate, or isolates distinct to those obtained through CNS samples, in 10 children. All *Streptococcus anginosus* Group (SAG) isolates were sensitive to penicillin (n=22). All *S. aureus* (n=8) was methicillin sensitive. Antibiotic sensitivities for anaerobes were not routinely assayed. Numbers refer to children with a given bacteria isolated. SDE, subdural empyema; EDE, extradural empyema.
